# Sorption behavior of *Arachis hypogaea* shells against Ag^+^ ions and assessment of antimicrobial properties of the product

**DOI:** 10.1007/s11356-020-08464-2

**Published:** 2020-03-25

**Authors:** Paweł Staroń, Krzysztof Pszczółka, Jarosław Chwastowski, Marcin Banach

**Affiliations:** Department of Engineering and Chemical Technology, Cracow University of Technology, 24 Warszawska St., 31-155 Cracow, Poland

**Keywords:** Ion silver, NanoAg, Antimicrobial activity, Sorption, Kinetic

## Abstract

The paper presents sorption behavior of *Arachis hypogaea* shells towards silver ions and possibility of their use as antimicrobial product. During the modification process of the natural sorbent, equilibrium tests were carried out. Moreover, the possibility of obtaining biocomposite *Arachis hypogaea* shells/nAg has been determined, and its antimicrobial properties have been evaluated. Additionally, sorption kinetics has been calculated. In the last step, silver ions were desorbed. The conducted equilibrium tests allowed to adjust the sorption isotherm model and determine the sorption capacity of tested material. This process is best described by Freudlich’s isotherm, and the sorption capacity is equal to 12.33 mg/g. On the basis of kinetic studies, the chemical nature of this process has been proved (by choosing a pseudo-second order model for the sorption process). It has been confirmed that the obtained peanut shells modified with silver ions have antimicrobial properties. The tests allowed to obtain 100% inhibition of *Aspergillus niger* and ~ 98% *Escherichia coli*.

**Electronic supplementary material:**

The online version of this article (10.1007/s11356-020-08464-2) contains supplementary material, which is available to authorized users.

## Introduction

Disease-causing microorganisms surround us every day. Microbes can enter our body in many ways. One of them is through the contact with biologically polluted water. Currently, several water treatment technologies are used, such as chlorination, UV disinfection, membrane separation, and adhesion. In the literature, one can find a lot of information about the antibacterial properties of various substances. One of the leading is silver, which model of operation has been very well characterized and described. The literature says that the antibacterial activity of silver ions is directly proportional to their concentration, and even at very low concentrations shows high efficiency. Researchers have tried to modify substances in a variety of ways to give them antimicrobial properties. For example, modifications of activated carbon with Al_2_O_3_ and Zn(OH)_2_ to inhibit growth of *Escherichia coli* (Pal et al. 2006). Another example was the chemical modification of cellulose in order to give it inhibitory properties against *E. coli*, *Enterococcus faecalis*, and *Staphylococcus aureus* (Saravanan and Ravikumar 2015). Zhou et al. developed a zerovalent iron biochar composite with strong antimicrobial properties against *Escherichia coli* (Zhou et al. 2014).

It can be seen in the literature that researchers are looking for natural materials that could be used as natural sorbents. There is a huge variety among organic sorbents. It results directly from their availability and high efficiency. In addition, biosorbents do not emit additional pollutants and are easy to dispose of, which is their great advantage. A large group of materials in which there is a high chance of their development, in the form of biosorbents, is agricultural wastes and parts of plants that cannot be used in the food industry. These materials are highly efficient, cheap, and renewable source of the biomass. The yield of a given type of sorbent depends on capacity, affinity, and its physicochemical nature. Plant wastes are tested both in their natural form and chemically and thermally modified to increase their sorption capacity. Among them are also bio-waste from food production such as coconut fibers (Chwastowski et al. 2017), apple peelings (Enniya et al. 2018), residues from brewing processes (Baylie 2016), or peanut shells (Gong et al. 2011).

Peanut (*Arachis hypogaea*) is a cultivated plant that belongs to the bean family. Currently, it is grown all over the world, and the main producers are India, China, the USA, and countries in Sub-Saharan Africa (Stalker et al. 2016). In 2016, almost 44 million tons of nuts were produced, of which approximately 25% of the total weight are shells (Perea-Moreno et al. 2018). Peanut is a geocarpic plant, which affects the structure of the shell, which consists of cellulose (45%), lignin (36%), hemicellulose (5.5%), and many biologically active compounds such as proteins and fats (Sareena et al. 2014).

The purpose of the work was to develop a method of modification of natural sorbent with metal ions by sorption, which will allow to obtain material with antimicrobial properties. The tested metal was silver among others due to the fact that its ions and compounds are highly toxic to microorganisms, including highly biocidal activity on 12 species of bacteria, for example *E. coli* (Kim et al. 2007). Silver is still used in a wide range of medical applications due to its antibacterial activity and low toxicity to human cells. Examples of silver applications include the use of silver-impregnated polymers to prevent bacterial growth on medical devices such as coils or heart valves. Silver is also used as a disinfectant in water systems. The killing effect of silver is associated with its rapid interaction with sulfhydryl groups on the surface of microorganisms, replacing hydrogen atoms, which causes the formation of an S - Ag bond. The result is complete blocking of respiration and electron transfer, which makes it difficult for microorganisms to induce effective rescue mechanisms (Mijnendonckx et al. 2013).

The process of surface modification of peanut shell was carried out in a dynamic system. The maximum sorption capacity of the material in relation to the tested metal as well as sorption mechanism was determined. Due to the presence of polyphenols in peanut shells and their reducing and stabilizing ability through steric stabilization, additional tests were carried out to obtain nanostructured silver on the surface of peanut shells analogously to the tests carried out in a previous work (Staroń et al. 2017). Microbiological tests have shown that the obtained material is characterized by inhibitory effect on *Aspergillus niger* and *Escherichia coli*.

## Materials and methods

### Materials

Peanut shells (PS) were obtained from peanuts bought in a commercial store. The shells were ground in a mill to obtain a homogeneous fraction. The material was sieved to obtain uniform size and then dried in an oven at 70 °C to remove any moisture. The chemical reagents used in the study were from Sigma-Aldrich and were of high purity. All solutions used in the study were prepared with the use of demineralized water.

### Surface modification

The first stage of peanut shell surface modification was to check, based on literature reports, three metal salts with biocidal properties: silver nitrate (V), copper (II) sulfate, and zinc nitrate (V).

Surface modification of nut shells for all salts was carried out as follows: 0.2-g ground peanut shells were weighed in the polypropylene containers, and 20-cm^3^ solution of a given salt at a concentration of about 150 mg/dm^3^ and at given pH was added. A series of solutions was prepared, respectively, at pH 1, 2, 3, 4, and 5 and natural for each substance. The solutions were stabilized with acids corresponding to the anions of the given salt, i.e., HNO_3_ and H_2_SO_4_. The sorption process was carried out for 1 h. After the modification process, shell was separated from the solutions, and the content of a given ion in the obtained filtrate was determined. Based on Eq. (1), the sorption capacity at equilibrium was determined

1 $$ {\mathrm{q}}_{\mathrm{e}}=\frac{\left({\mathrm{C}}_0-{\mathrm{C}}_{\mathrm{e}}\right)}{\mathrm{w}}.\mathrm{V} $$

where

- q_e_ – mass of adsorbed metal at equilibrium (mg/g),
- C_0_ – initial ion concentration (mg/dm^3^),
- C_e_ – ion concentration in equilibrium (mg/dm^3^),
- V – volume of solution (dm^3^),
- w – peanut shell mass (g).

Modified peanut shells after drying to the constant weight (70 °C, 24 h) were subjected to preliminary microbiological tests, which allowed to determine the biocidal properties of the obtained material and finally select the metal ion for proper tests (the highest sorption capacities were observed for solutions with a pH of 5 and without any modification). This is consistent with the theory that as the concentration of cations increases, the number of free active centers decreases as they bind together (Zabochnicka-Światek and Krzywonos 2014). Preliminary microbiological tests showed that silver ions were characterized by the best biocidal properties. Results are presented in table S1.

### Sorption process

The sorption process was carried out in a dynamic bed system. The effect of time on achieving equilibrium for various silver ion concentrations was investigated. Solutions with a concentration of (Ag) 100, 200, 300, 400, and 500 mg/dm^3^ were prepared. To 0.5 g of peanut shells, 50 cm^3^ of a solution of appropriate silver concentration was added, followed by sorption for 0.5, 1, 1.5, 2, 3, and 5 min on a magnetic stirrer. After the sorption process was completed, the sorption capacity was calculated using Eq. (1). All tests were repeated three times, and the results were averaged.

### Statistical analysis of the fitted models

The estimation of parameters in equilibrium and kinetic models was made using the nonlinear regression method. Statistical analysis was performed in the 12th version of STATISTICA Statsoft®. The quality of the results obtained has been verified by the determination coefficient (*R*^2^) and the average relative error (ARE) (Marques et al. 2018, 2019)

2 $$ {R}^2=1-\frac{\sum \limits_1^n{\left({\mathrm{q}}_{\mathrm{exp}}-{\mathrm{q}}_{\mathrm{pred}}\right)}^2}{\sum \limits_1^n{\left({\mathrm{q}}_{\mathrm{exp}}-\overline{{\mathrm{q}}_{\mathrm{exp}}}\right)}^2} $$

3 $$ ARE=\frac{100}{n}\sum \limits_1^n\frac{\mid {\mathrm{q}}_{\mathrm{exp}}-{\mathrm{q}}_{\mathrm{pred}}\mid }{{\mathrm{q}}_{\mathrm{exp}}} $$

where

- *n* – number of experimental points,
- q_exp_ – experimental sorption capacity (mg/g),
- q_pred_ – predicted sorption capacity (mg/g),
- $\overline{q_{exp}}$ – the mean of experimental sorption capacity (mg/g).

### Equilibrium studies

Obtained results allowed modeling of equilibrium parameters based on four sorption isotherms.

The first model was the Langmuir isotherm, which describes the formation of a monolayer on the adsorbent surface. It can be successfully used for diluted electrolyte solutions. A simple equation was used for the calculations (Wu et al. 2010):

4 $$ {\mathrm{q}}_{\mathrm{e}}=\frac{{\mathrm{q}}_{\mathrm{m}}{\mathrm{K}}_{\mathrm{L}}{\mathrm{C}}_{\mathrm{e}}}{1+{\mathrm{K}}_{\mathrm{L}}{\mathrm{C}}_{\mathrm{e}}} $$

where

- q_e_ – equilibrium sorption capacity (mg/g),
- q_m_ – maximum sorption capacity (mg/g),
- C_e_ – equilibrium concentration (mg/dm^3^),
- K_L_ – Langmuir’s constant (L/mg).

The Freundlich isotherm model is an empirical isotherm and is used to describe sorption properties for heterogeneous adsorbent surfaces (Atkins 2003). The theory assumes that sorption processes are reversible, imperfect, and multilayered. The form of the isotherm in the form of a simple equation was used in the calculations (Baláž et al. 2005):

5 $$ {\mathrm{q}}_{\mathrm{e}}={\mathrm{K}}_{\mathrm{F}}{\mathrm{C}}_{\mathrm{e}}^{\raisebox{1ex}{$1$}\!\left/ \!\raisebox{-1ex}{$n$}\right.} $$

where

- K_F_ – Freundlich constant (mg^1-(1/*n*)^(dm^3^)^1/*n*^g^−1^),
- *n* – heterogeneity parameter.

The Temkin isotherm model is based on the hypothesis that the heat of adsorption is due to the interaction of the adsorbate and decreases linearly. The process is characterized by an even distribution of energy that reaches a certain maximum. The model is expressed by the formula (Mousa et al. 2016):

6 $$ {\mathrm{q}}_{\mathrm{e}}={\mathrm{BlnK}}_{\mathrm{T}}{\mathrm{C}}_{\mathrm{e}} $$

7 $$ \mathrm{B}=\frac{\mathrm{RT}}{{\mathrm{b}}_{\mathrm{t}}} $$

where

- K_T_ – binding equilibrium constant corresponding to the maximum binding energy (dm^3^/g),
- B – constant associated with the heat of sorption (J/mol),
- R – gas constant (8.314 J mol/K),
- T – process temperature (K),
- b_t_ – Temkin constant isotherms.

The Dubinin-Radushkevich (D-R) isotherm model is commonly used to express the mechanism of sorbent adsorption on heterogeneous porous structures. The formula was used to calculate the parameters (Dada et al. 2012):

8 $$ {\mathrm{q}}_{\mathrm{e}}={\mathrm{q}}_{\mathrm{d}}\exp \left(-{K}_{ad}{\varepsilon}^2\right) $$

9 $$ \upvarepsilon =\mathrm{RTln}\left(1+\frac{1}{{\mathrm{C}}_{\mathrm{e}}}\right) $$

where

- q_d_ – theoretical maximum sorption capacity (mg/g),
- K_ad_ – Dubinin-Radushkevich’s isotherms associated with sorption energy (mol^2^/J^2^),
- ε – Polanyi’s constant.

One of the additional parameters is the average sorption energy expressed in J/mol:

10 $$ E=\frac{1}{\sqrt{2{\mathrm{B}}_{\mathrm{d}}}} $$

### Kinetic models

One of the most important parameters affecting the effectiveness of the adsorption process is the contact time between the adsorbent and the adsorbate. The influence of contact time and the kinetic behavior of the sorption process were investigated. Sorption capacity was measured for various initial concentrations and various time intervals from addition of nut shell to ion solution. The kinetics of the process was calculated on the basis of four kinetic models.

A pseudo-first kinetic model in which it is assumed that the rate of adsorption of metal ions over time is directly proportional to the difference between the mass of adsorbed ions at equilibrium and the mass of adsorbed ions at the time of sampling. The nonlinear relationship of this model is shown in Eq. 11 (Cheung et al. 2000):

11 $$ {\mathrm{q}}_{\mathrm{t}}={\mathrm{q}}_1\left(1-\exp \left(-{\mathrm{k}}_1\mathrm{t}\right)\right) $$

where

- q_1_ – estimated sorption capacity (mg/g),
- q_t_ – sorption capacity in time t (mg/g),
- k_1_ – pseudo-primary constant (1/min),
- t – time (min).

The pseudo-secondary kinetic model assumes the pseudochemical nature of the sorption process. The driving force is the difference between the equilibrium sorption capacity and the mass of adsorbed ions at the time of stopping the process. The speed of the process is proportional to the square of the driving force. Equation 12 represents the described model (Kumar 2006).

12 $$ {\mathrm{q}}_{\mathrm{t}}=\frac{\mathrm{t}}{\left(1/{\mathrm{k}}_2{\mathrm{q}}_2^2\right)+\left(\mathrm{t}/{\mathrm{q}}_2\right)} $$

where

- q_2_ – estimated sorption capacity (mg/g),
- k_2_ – pseudo-second model constant (g/mg min).

Elovich’s model is a heterogeneous kinetic equation that describes the adsorption of gases on solid surfaces. The theory assumes multilayer adsorption, and the number of sites increases exponentially with sorption. Elovich’s model is shown in Eq. 13 (Marques et al. 2019).

13 $$ {\mathrm{q}}_{\mathrm{t}}=\frac{1}{a}\ln \left(1+\mathrm{abt}\right) $$

where

- a – desorption constant of Elovich model (g/mg),
- b – represents the initial velocity when q_t_ = 0 (mg/g min).

The Weber-Morris model is the theory of intramolecular diffusion, which assumes that the phase determining the sorption process is the sorbate diffusion effect. Intermolecular diffusion is constant and its direction is radial. Equation 14 represents the described model (Svilović et al. 2010).

14 $$ {\mathrm{q}}_{\mathrm{t}}={\mathrm{K}}_{\mathrm{id}}\sqrt{\mathrm{t}}+I $$

where

- K_id_ – molecule diffusion rate constant (mg/g min^0.5^),
- I – fixed value parameter proportional to the boundary layer.

### Desorption

The process of desorption of silver ions from the surface of peanut shells was carried out as follows:

1. A total of 250 cm^3^ of 500 mg/dm^3^ solution (Ag) was prepared. Then, 2-g peanut shells were added to the solution and placed on a magnetic stirrer. The process was carried out at 20 °C for 60 min. After sorption, the whole probe was filtered under reduced pressure, a filtrate sample was taken for analysis for the presence of silver ions, and the material was washed with deionized water and dried to constant weight (70 °C, 24 h).
2. The desorption process was carried out (the eluents used for the silver ion desorption were as follows: H_2_O and citric acid, acetic acid, sodium hydroxide at concentration of 0.5 mol/dm^3^).

After drying, 0.25 g of modified peanut shells and 50 cm^3^ of each of the eluents were taken into a beaker, and then placed on a magnetic stirrer. The process was carried out at 20 °C for 60 min. The samples were filtered under reduced pressure, and the filtrates were analyzed for silver ion content. The desorption process was repeated three times, and the results were averaged.

The degree of desorption was determined based on the following equation:

15 $$ {\mathrm{R}}_{\mathrm{des}}=\frac{{\mathrm{A}}_{\mathrm{sol}}}{{\mathrm{A}}_{\mathrm{sor}}}100\% $$

where

- A_sol_ – mass of silver ions in the filtrate after the desorption process (mg),
- A_sor_ – the mass of silver ions remained on the sorbent after the desorption process (mg).

### Microbiological tests

*Aspergillus niger* and *Escherichia coli* were used in the microbiological study.

*Aspergillus niger* is an organism known as the black *Aspergillus* and is commonly found all over the world. At the first stage of its development, it forms characteristic white colonies. During development, it turns yellow, and then dark brown conidiophores appear. The most favorable places for the development of black *Aspergillus* are those in which humidity appears and a temperature is in the range between 6 and 47 °C. By attacking food, this organism produces aflatoxin, which is dangerous to humans (Dijksterhuis 2013).

*Escherichia coli* is the predominant facultative anaerobe of the human and other mammal’s colonic flora. Several different *E. coli* strains cause diverse intestinal and extra intestinal diseases by the virulence factors which can affect a wide range of cellular processes (Kaper et al. 2004). Infections due to pathogenic *E. coli* may be limited to the mucosal surfaces or can disseminate throughout the body (Nataro and Kaper 1998).

A sterilized 10-cm^3^ YPD standard medium was prepared. It included yeast extract, casein peptone, sucrose, and agar. The medium was spread on a Petri dish with a diameter of 8 cm. Then, after solidification, 50 mg (for *A. niger*) or 200 mg (for *E. coli*) of the tested material was added to each sample, and 50 μl of suspension containing *Aspergillus niger* cells or a specified number of *Escherichia coli* cultures was provided. In the next step, the preparations were evenly distributed over the surface of the entire medium using a smoothing pad. The cultures prepared in this way were placed in an incubator at ~ 27 °C. After 24 and 48 h, photographs were taken to assess the growth of microorganisms relative to selected materials.

## Results and discussion

**Fig. 1:**
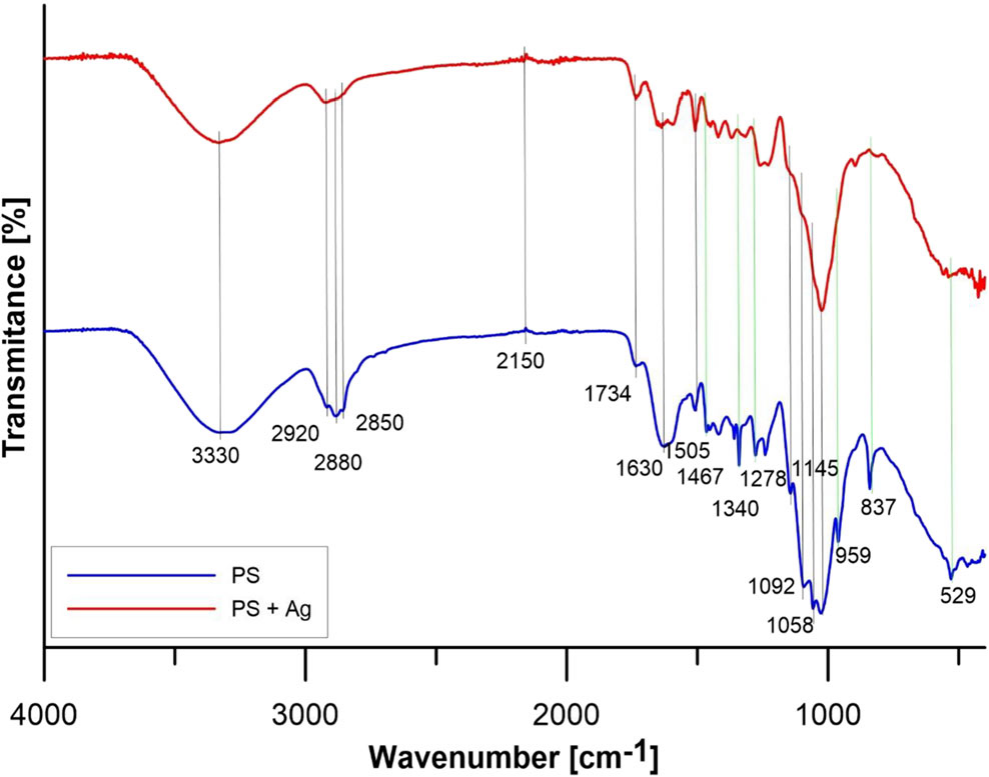
FT-IR spectrum of peanut shells before and after sorption

The conducted instrumental methods allowed to characterize peanut shells before and after the sorption process. Fourier transform infrared spectroscopy (Nicolet iS5 with ATR iD7 from Thermo Scientific) was performed (Fig. 1). The study identified characteristic peaks at a wavenumber of 3350–3450 cm^−1^ corresponding to the vibrations of cellulosic OH^−^ groups (Prabhakar et al. 2015); 1630 cm^−1^ corresponds to stretching vibration C=O (Villena et al. 2000); a strong band at 1058 cm^−1^ corresponds to C-O stretching, while corresponding bending bonds are observed at 837 cm^−1^ (Wahab et al. 2012); other bands in the 2920–2850 cm^−1^ range may represent CH_2_ asymmetric vibrations and C-H stretching vibrations (Bhaduri et al. 2016); the band at 1734 cm^−1^ represents carbonyl tensile vibrations (-C=O) (Manoj Kumar Reddy et al. 2013); the band at 1508 cm^−1^ may be associated with the presence of phenol groups and aromatic rings (Smidt and Meissl 2007); 1467 and 1278 cm^−1^ possibly due to C-O, C-H, or C-C stretching vibrations (Georgin et al. 2016); 1340 cm^−1^ aliphatic C-H stretching in methyl and phenol alcohols (Taşar et al. 2014); 1220–1020 cm^−1^ (generally weak) area is usually responsible for CN binding of aliphatic amine compounds; vibrations in the NH position occur in the 910–660 cm^−1^ band; weak bands in the frequency range 600–400 cm^−1^ are caused by vibration deformation CNC (Gunasekaran et al. 2006); the peak at 2150 cm^−1^ represents CO_2_ that always remains in the environment (Singh and Vaish 2018).

In addition, it can be seen that for the material after sorption of silver ions, there were fading signals that appeared in the spectrum of the material before sorption. These peaks were present at a wavenumber of approximately 1467 cm^−1^, 1340 cm^−1^, 1278 cm^−1^, 959 cm^−1^, 837 cm^−1^, and 529 cm^−1^ (marked with green lines in the figure). The above changes in FT-IR spectra indicate that among others nitrile and carbonyl groups and C-H bond are involved in silver ion adsorption. Silver creates among others complexes on the surface of peanut shell (OuYang et al. 2014).

**Fig. 2:**
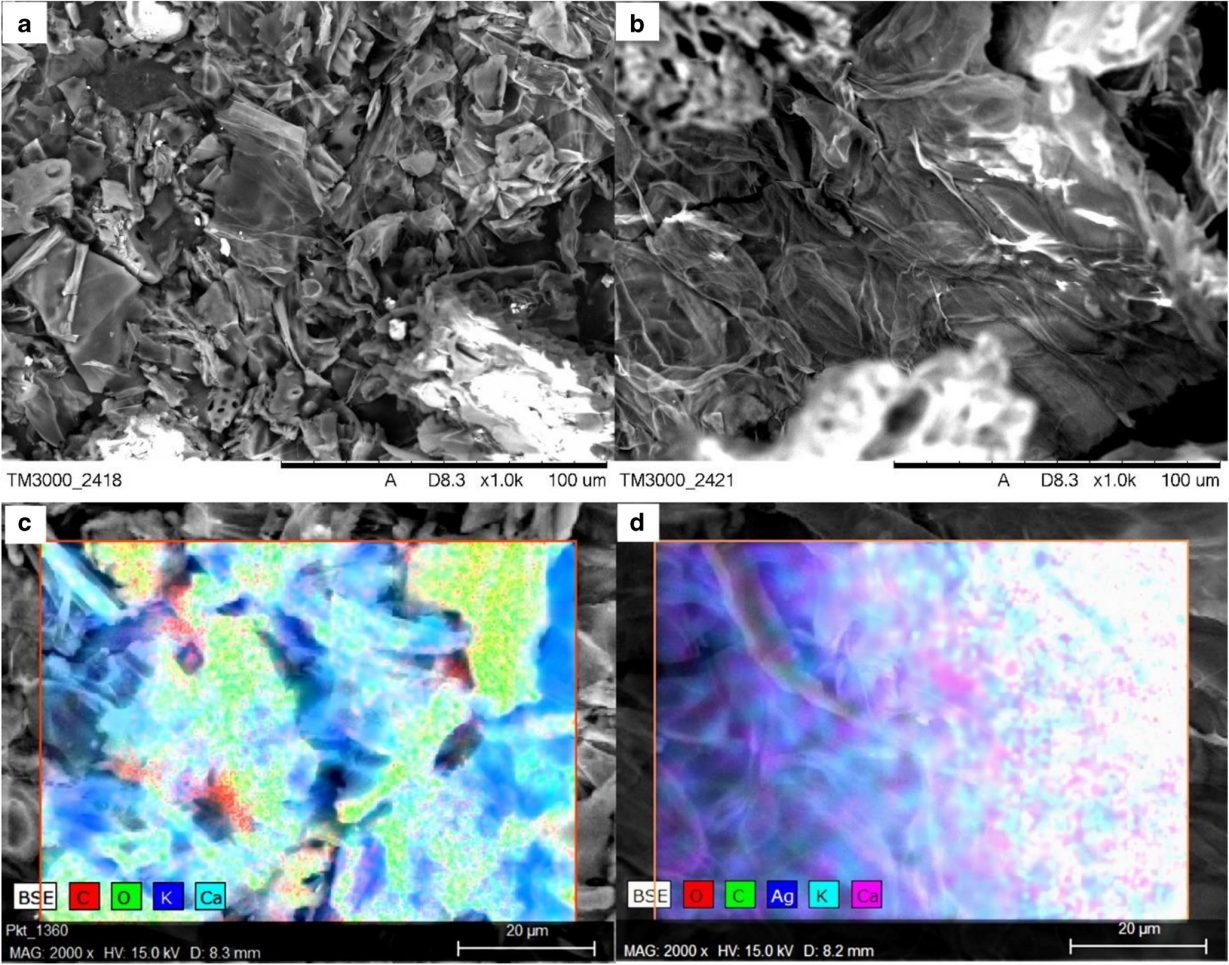
SEM-EDS micrograph of peanut shells. **a**, **c** Before sorption. **b**, **d** After sorption

SEM micrograph (Fig. 2) presents the surface of the material before and after the silver ion sorption process (Hitachi TM-3000 equipped with an X-ray microanalyzer EDS). Large fragmentation of peanut shells and diversified particle size can be seen, moreover, after the sorption process (Fig. 2b), a more structured material can be observed. Figure 2c and d present the analysis of the chemical composition of the surface of materials before (c) and after (d) sorption. As expected, before the sorption process, next to carbon and oxygen, metals from groups I and II of the periodic table have the largest share, and after the sorption process, their share decreases in favor of silver, which is bound to the sorbent surface.

### Influence of initial concentration and time on silver ion sorption

**Fig. 3:**
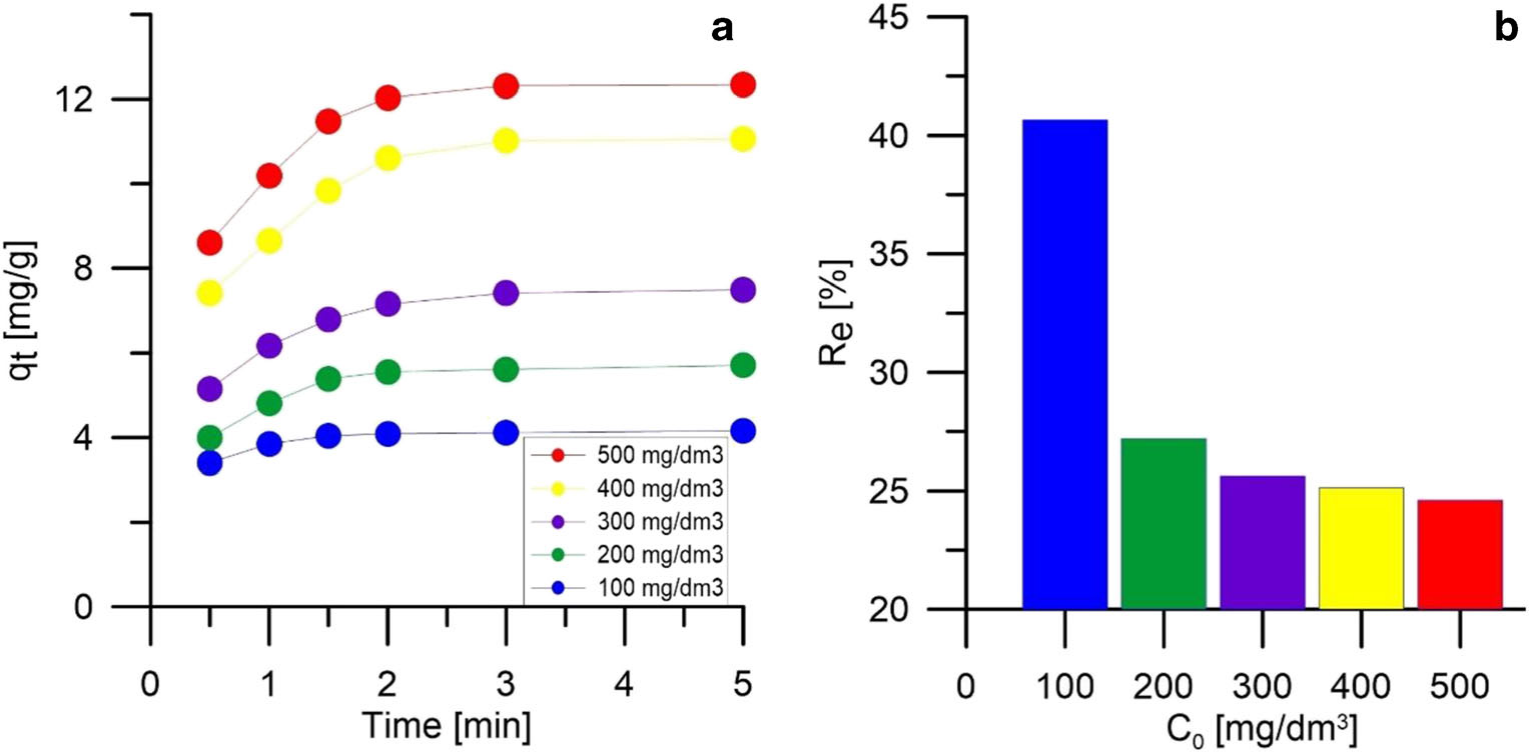
**a** Sorption capacity of peanut shells in time depending on the initial concentration. **b** The degree of silver ion removal by peanut shells

Figure 3a shows how the sorption capacity of the material changed depending on the duration of the process and the degree of silver ion removal by the material being tested. It can be seen that the process takes place very quickly and is close to equilibrium after only 3 min. The largest increase in sorption capacity is observed in 2 min, after which the number of active places on the surface of peanut shells is reduced and the equilibrium between sorbent and sorbate is established. In addition, an increase in sorption capacity was observed with an increase in initial concentration, which is the result of an increase in the mass transfer driving force necessary to overcome the resistance of ion mass transfer between sorbent and sorbate. In addition, increasing the initial concentration of silver ions increases the number of collisions between them and the active sites of the sorbent (Al-Rub et al. 2004). Figure 3b shows the effect of the initial concentration of silver ions on the degree of their removal from the solution. The value of the degree of removal decreases with the increase of the initial concentration of silver ions, which is the result of a change in the ratio of the number of ions in the solution to the number of active sites on the sorbent surface (constant sorbent mass). The impact of the number of collisions resulting in an increase in sorption capacity means that the difference in the degree of silver ion removal between the concentration of 100 and 200 mg/dm^3^ is much higher (~ 13 p.p.) than between the concentration of 200–500 mg/dm^3^ (0.5–1.7 p.p.).

### Equilibrium studies

**Fig. 4:**
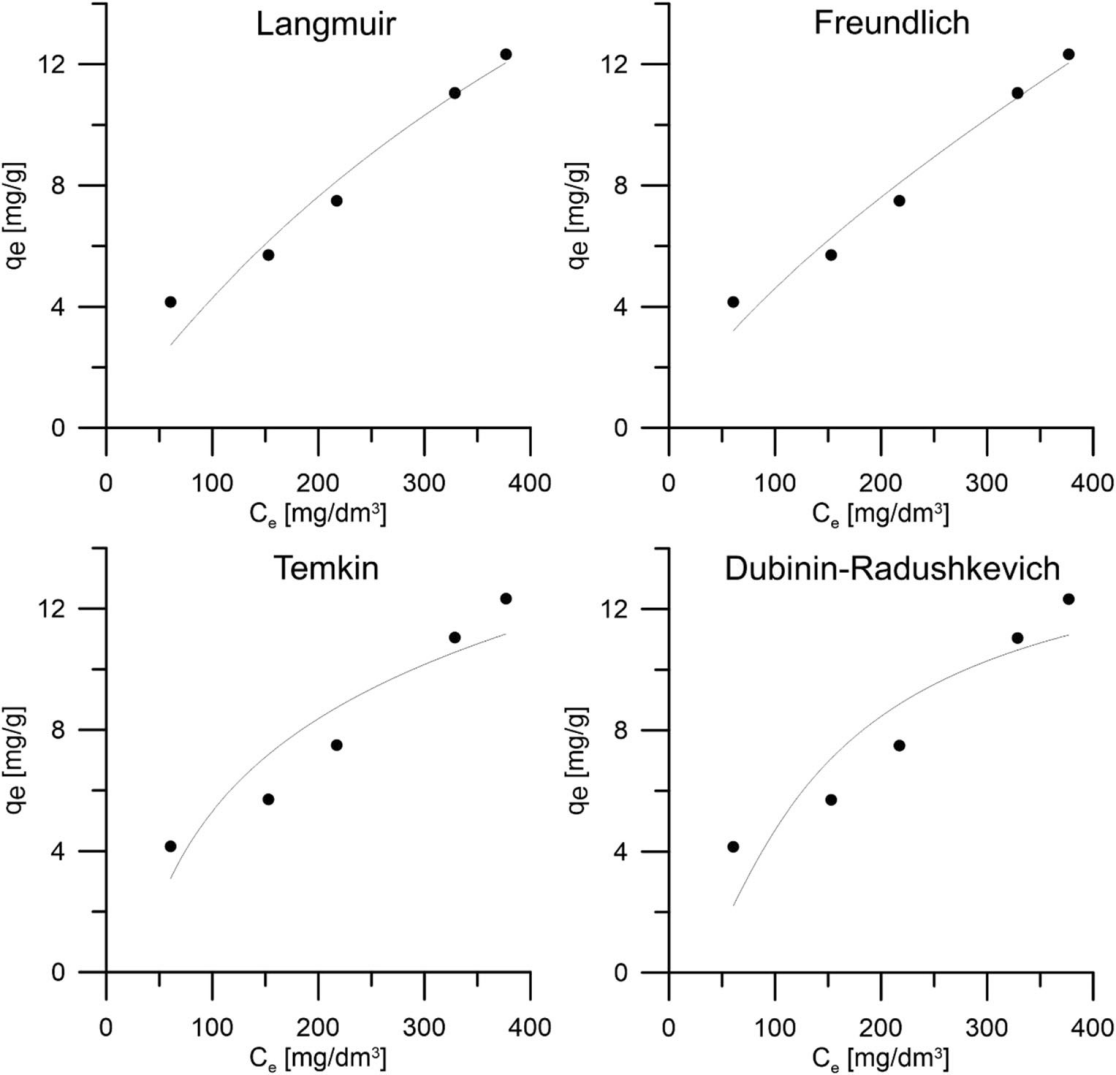
Graphic representation of sorption isotherms for the process of silver ion adsorption on peanut shells

Studies on achieving equilibrium by sorption systems allow modeling of processes and explain sorption mechanisms. To this end, equilibrium data for four isotherm models was analyzed: Langmuir, Freundlich, Temkin, and D-R. Figure 4 graphically shows the matches of sorption models, and Table 1 presents the values of parameters of isothermal models. By means of nonlinear regression, the correlation coefficient *R*^2^ and the average relative error (ARE) was determined, which shows that the best model that describes the ion sorption model on peanut shells is Freundlich’s isotherm. In the Freundlich model, the sorbent adsorbs solute relatively easily when the 1/*n* parameter is less than 1. The calculated coefficient 1/*n* is ~ 0.7, which indicates that peanut shells are suitable for silver ion adsorption (Mu and Sun 2019). A value of 1/*n* < 1 indicates that sorption capacity is slightly suppressed at lower equilibrium concentrations. Freundlich’s isotherm does not predict the saturation of peanut shells with silver ions; therefore, it predicts mathematically infinite surface coverage indicating multilayer adsorption on the surface (Erdem et al. 2004). In addition, if *n* is from one to ten (*n* = 1.39), this indicates a favorable sorption process (Dada et al. 2012).

**Table 1:** Parameters of sorption isotherms models for the process of silver ion sorption on peanut shells

Isotherm model	Parameters			
**Langmuir**	**ARE (%)**	***R***^**2**^	**q**_**m**_**(mg/g)**	**K**_**L**_**(dm**^**3**^**/mg)**
	10.77	0.9430	34.728	0.001409
**Freundlich**	**ARE (%)**	***R***^**2**^	**K**_**F**_**(mg**^**1-(1/n)**^**(dm**^**3**^**)**^**1/*n***^**g**^**−1**^**)**	**1/n**
	8.88	0.9652	0.164641	0.72355
**Temkin**	**ARE (%)**	***R***^**2**^	**K**_**T**_**(dm**^**3**^**/g)**	**B**
	16.42	0.8659	0.033247	4.4181
**D-R**	**ARE (%)**	***R***^**2**^	**K**_**ad**_**(mol**^**2**^**/kJ**^**2**^**)**	**q**_**d**_**(mg/g)**
	20.48	0.8113	0.048447	15.246

### Kinetic studies

With a view to understanding the mechanisms of silver ion sorption on peanut shell surfaces, research was conducted on the effect of time and initial concentrations. The speed of sorption can be limited by many factors, including diffusion of sorbate to the surface of the sorbent, diffusion on the surface, diffusion into the pores, and interaction of the sorbate with active centers.

Analyzing the results of sorption of silver ions on peanut shells, it can be observed that as the initial concentration of sorbate increases, the equilibrium time between sorbate and sorbent lengthens. The shortest time to equilibrium was recorded for an initial concentration of 100 mg/dm^3^, which was 1.5 min, and for 200 mg/dm^3^, which was 2 min, and for the remaining concentrations, it was 3 min. In addition, it was observed that for the initial concentration, the smallest percentage increase in sorption capacity occurs between the first and last measurement. For a concentration of 100 mg/dm^3^, an increase of 20% was noted, and for other concentrations ~ 45%. This is due to the fact that at the lowest concentration, the amount of silver ions is so low that they fill the active sites very quickly. The few ions remaining in the solution no longer have such a high driving force, which is why the number of active collisions between them and sorbent is less effective (Al-Rub et al. 2004).

**Fig. 5:**
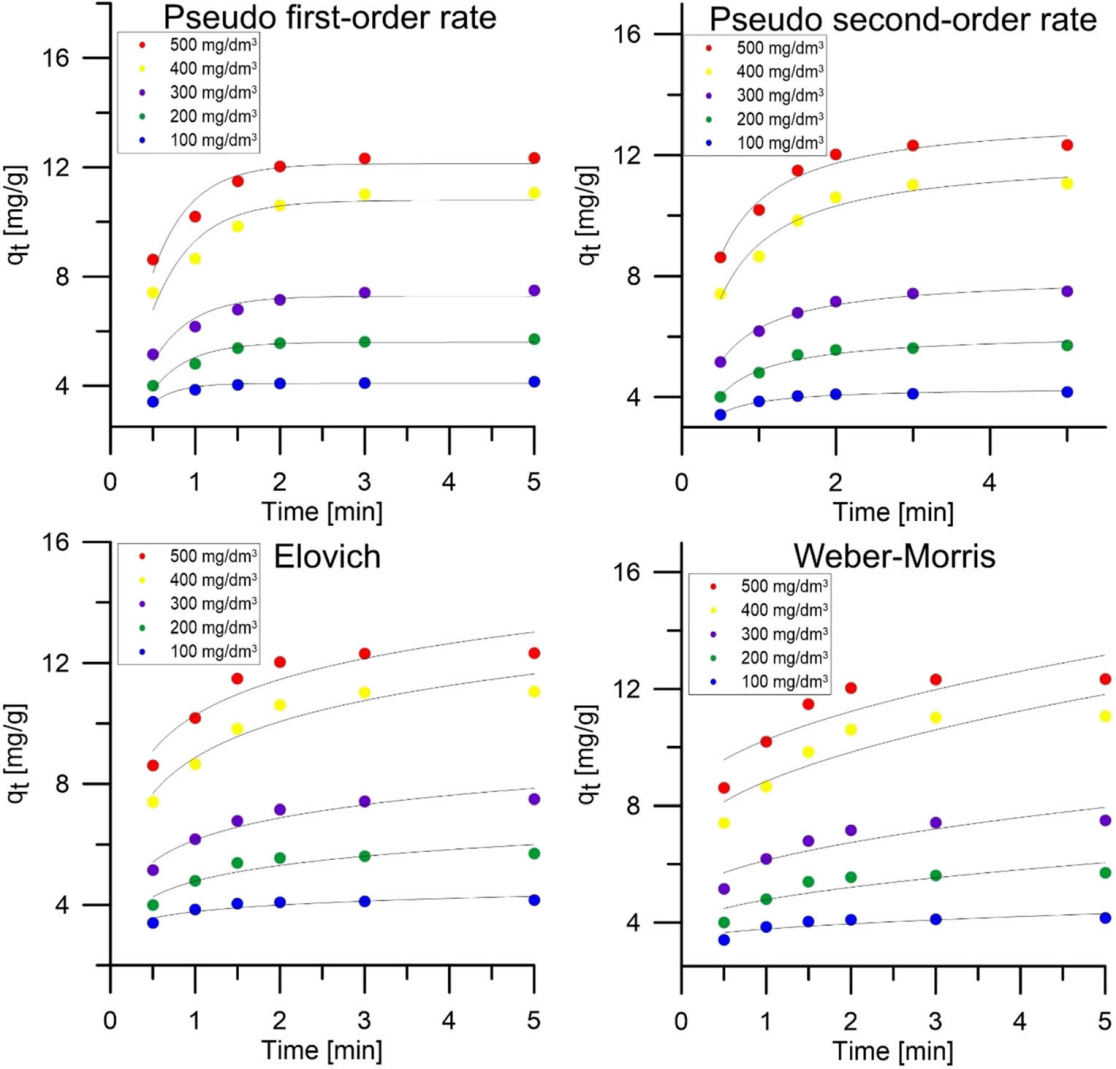
Graphic representation of sorption kinetic models for the process of silver ion adsorption on peanut shells

The following kinetic models were used in the work: the pseudo-first order model, the pseudo-second order model, the Elovich, and Weber-Morris model. A summary of kinetic models and their parameters is shown in Table 2 and in a graphical way in Fig. 5. Based on the calculated fit factor (*R*^2^) and the average relative error (ARE), it was found that the model that best describes the kinetics of silver ion sorption on the surface of peanut shells is the pseudo-second order model. At all analyzed initial concentrations, the second order model has *R*^2^ > 0.967. This adjustment allows to state that the sorption process is of a chemical nature. It is related to the interaction of valence forces by exchanging or dividing electrons between silver ions and biosorbent (Ho 2006).

**Table 2:** Parameters of sorption kinetic models for the sorption process silver ions on peanut shells

Kinetic model	Silver ion concentration C_0_ (mg/dm^3^)				
	100	200	300	400	500
Pseudo-primary model					
q_1_ (mg/g)	4.088	5.5981	7.2889	10.7888	12.1310
k_1_(min^−1^)	3.4618	2.3159	2.2079	1.9799	2.2218
*R*^2^	0.9509	0.9555	0.9265	0.8947	0.9323
ARE (%)	1.18	2.02	3.21	4.20	2.86
Pseudo-secondary model					
q_2_ (mg/g)	4.314	6.130	8.039	12.003	13.337
k_2_ (min^−1^)	1.8304	0.6360	0.4425	0.2548	0.2741
*R*^2^	0.9756	0.9707	0.9899	0.9676	0.9711
ARE (%)	0.97	1.78	1.02	2.22	1.75
Evolich’s model					
a (g/mg)	3.160	1.327	0.945	0.579	0.584
b (mg/g min)	48,393.5	431.4	352.8	291.0	686.9
*R*^2^	0.8217	0.8597	0.9180	0.9168	0.8783
ARE (%)	2.57	3.69	3.12	3.59	3.78
Weber-Morris model					
I	3.3512	3.7536	4.6635	6.4167	7.9019
K_id_	0.4263	1.0281	1.4700	2.4142	2.3520
*R*^2^	0.66688	0.715496	0.793309	0.804656	0.741112
ARE (%)	3.43	5.54	5.14	5.71	5.67

### Desorption

Table 3 presents the results of the silver ion elution analysis from modified peanut shells using organic and inorganic eluents. It can be observed that by far, the biggest influence on ion desorption has two organic acids: acetic (18.3%) and citric (20.8%). This was due to reactions between groups in acid molecules and silver ions. Citric acid together with acetic acid belongs to the group of organic compounds capable of forming various types of complexes and as strong chelating agents can lead to greater leaching of silver ions from the biosorbent into the solution (Shan et al. 2002; Wang et al. 2013). Due to the high bonding energy between peanut shell and silver ions (resulting from chemisorption), water (2.5%) removed silver ions from the sorbent surface to a negligible extent. The use of sodium hydroxide as the eluent (elution only ~ 2%) was intended to produce nanometric silver particles on the surface of the adsorbent in order to give it additional biocidal properties. The formation of silver nanoparticles is associated with the presence of compounds with reducing and stabilizing capabilities (through steric stabilization) such as polyphenols. Confirmation of obtaining nanoparticle silver on the sorbent surface was among other change of surface color to brown, which indicates the presence of silver in the form of nanoAg (Staroń et al. 2017).

**Table 3:** Silver ion desorption from modified peanut shells

Eluent – concentration (mol/dm^3^)	Silver content before desorption (mg/g)	Silver content after desorption (mg/g)	Degree of desorption (%)
H_2_O	13.83	13.48	2.52
Sodium hydroxide – 0.5	13.83	13.54	2.14
Acetic acid – 0.5	13.83	11.30	18.28
Citric acid – 0.5	13.83	10.95	20.84

### Antimicrobial studies

**Fig. 6:**
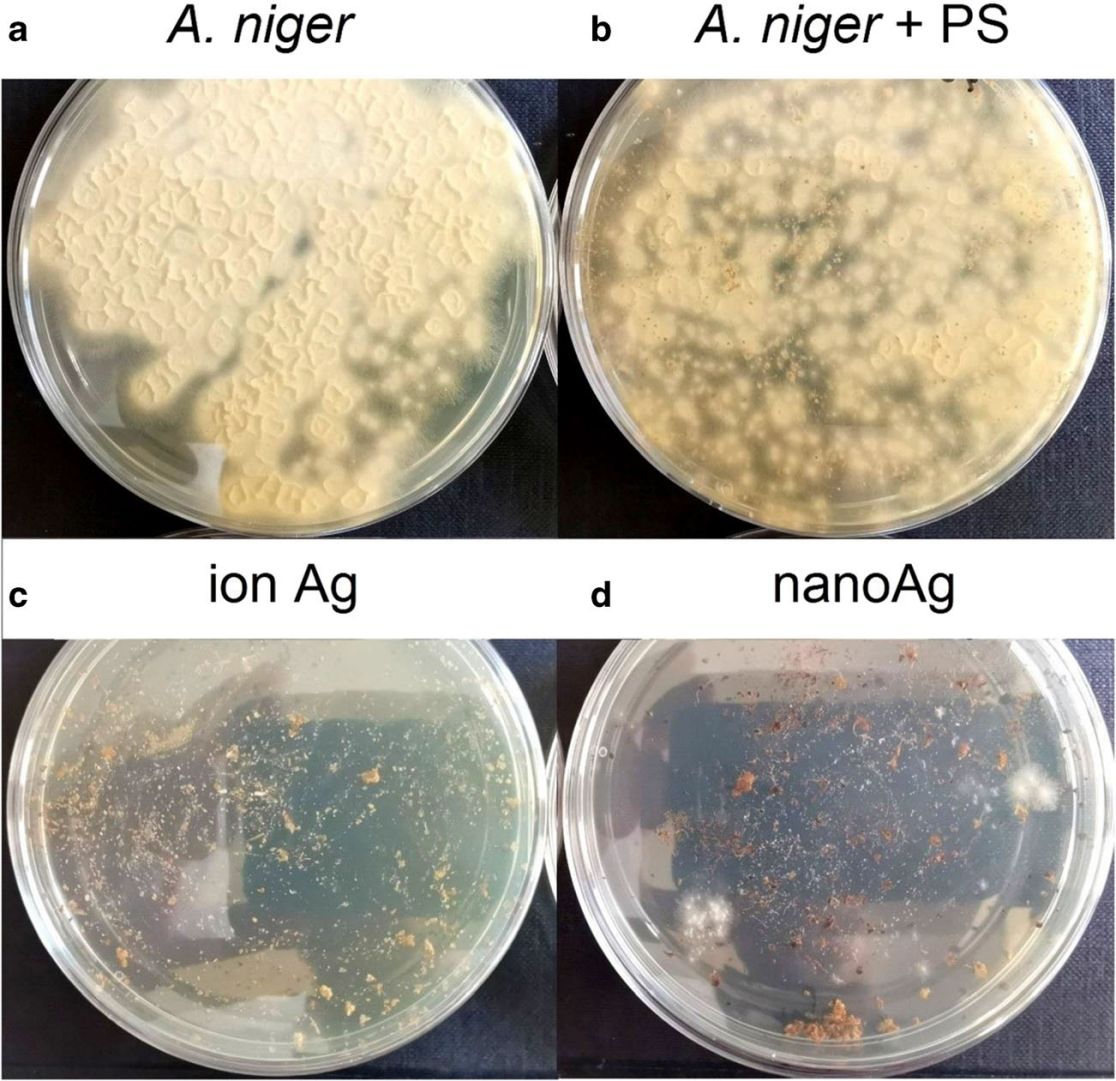
*Aspergillus niger* after 48 h of incubation. **a** Control sample, **b** Sample containing unmodified peanut shells, **c** Sample containing modified peanut shells with silver ions, **d** Sample containing modified peanut shells with silver nanoparticles

**Fig. 7:**
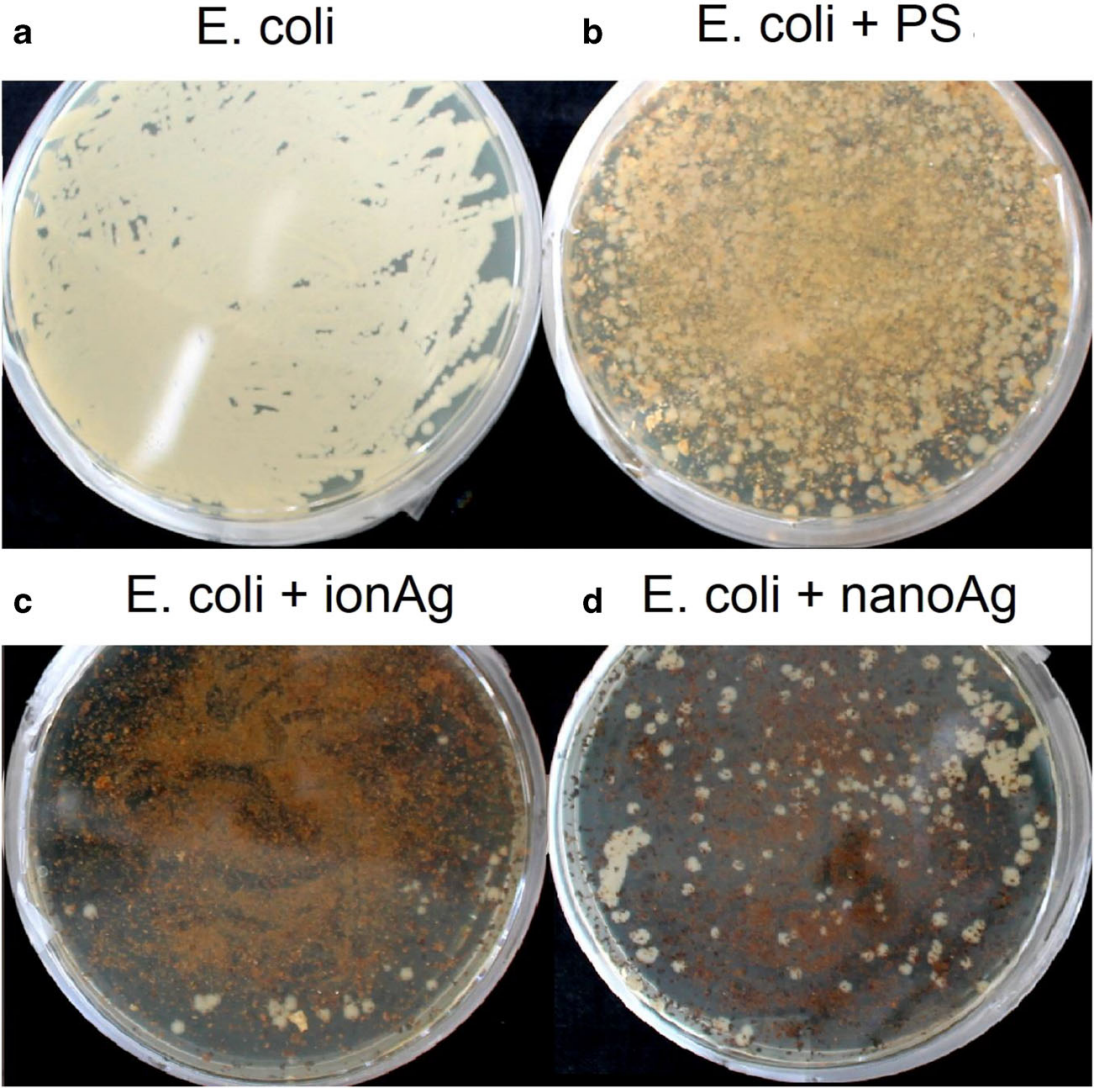
*Escherichia coli* after 24 h of incubation. **a** Control sample, **b** Sample containing unmodified peanut shells, **c** Sample containing modified peanut shells with silver ions, **d** Sample containing modified peanut shells with silver nanoparticles

Microbiological tests were carried out to determine the biocidal properties of the material. Figures 6 and 7 show the *Aspergillus niger* fungus, which was incubated for 48 h and the *Escherichia coli* bacteria incubated for 24 h. Table 4 shows their percentage of growth and inhibition. Figure 6a (control sample containing *Aspergillus niger*) and Fig. 6b (sample containing unmodified shells and *Aspergillus niger*) show the growth of the fungus after the second day by 70.9% (a) and 96.9% (b). Figure 7a and b show the growth of *Escherichia coli*, followed without and together with peanut shells. Bacteria growth of 99.1% (a) and 79.3% (b) was observed, which results in the ability to inhibit the growth of peanut shells by 20%. On this basis, it can be concluded that peanut shells not subjected to surface modification process are a source of carbon for the *Aspergillus niger* fungus, while for *Escherichia coli*, they have a biocidal effect. In the remaining two samples containing modified peanut shells with silver ions (c), no fungal growth was observed (Fig. 6) and a slight (~ 1.4%) growth of bacterial colonies was noted (Fig. 7), while for peanut shells with silver nanoparticles (d), an increase of 12.0% in *A. niger* was observed and 11.3% of *E. coli*.

**Table 4:** Growth and inhibition results for the organism *Aspergillus niger* and *Escherichia coli*

Sample	*Aspergillus niger*		*Escherichia coli*	
	Growth 48 h	Inhibition 48 h	Growth 24 h	Inhibition 24 h
	(%)			
A	70.9	-	99.1	-
B	96.9	− 36.7	79.3	20.1
C	0.0	100.0	1.4	98.6
D	12.0	87.6	11.3	88.6

On this basis, it can be concluded that the material containing silver in ionic form has better biocidal properties. Other researchers have come to similar conclusions. They showed that a given amount of silver ions is much more toxic to the cells of microorganisms than the same amount of silver in the form of silver nanoparticles. It is generally believed that the biological effect of silver is due to free silver ion (Choi et al. 2008; Greulich et al. 2012).

For ionic silver, 100% growth inhibition was observed for *Aspergillus niger* and 98.6% for *Escherichia coli*. This is due to the interaction of ions with the bacterial and fungal membrane, which leads to the separation of the cytoplasm from the cell wall and in the end to the cell death. Silver ions have been shown to enter bacterial cells within 30 min of exposure and react with cytoplasmic components (Kędziora et al. 2018). This was confirmed by, among others, Jung et al. in their research. Researchers have observed the effect of silver ion concentration on the reduction of *E. coli*. The number of *E. coli* was reduced from the inoculum size (10^5^ CFU/cm^3^) to the limit of detection (< 20 CFU/cm^3^) in 30 min at a concentration of 0.2 mg/dm^3^ silver ions (Woo et al. 2008). The results obtained related to *A. niger* growth inhibitory activity are similar to those obtained by other researchers. Pinto et al. conducted research on the effects of nanoparticle and ionic silver on the antifungal activity of composite transparent films against *Aspergillus niger*. Based on the results obtained, he found that a higher antifungal activity (FGI = 61%) was characterized by a composite containing silver in ionic form, compared with a composite containing nanoAg (FGI = 45%) with comparable silver content. This result seems to confirm that *A. niger* growth inhibition is associated with a mechanism involving the interaction of the fungus with cationic silver (Pinto et al. 2013).

## Conclusion

As a result of the actions carried out, the goal was to modify the natural sorbent to give it antimicrobial properties. The FT-IR and SEM-EDS tests confirmed the presence of silver on the surface of the material. The obtained material had a sorption capacity of 12.33 mg/g in relation to silver ions. The process of sorption of silver ions occurs according to the Freundlich sorption isotherm model as evidenced by the coefficient *R*^2^ = 0.94. Additionally, the calculated parameter 1/*n* indicates favorable sorption conditions, which was confirmed by the calculation of the separation factor *R*_L_ (all values below 1). The kinetics of the silver ion sorption process on peanut shells fits perfectly into the pseudo-second model for which *R*^2^ > 0.99. Based on the model, we can conclude that the stage limiting the process is chemisorption. The best eluents that leach silver ions are light organic acids, such as acetic or citric, for which the desorption percentage is 18.3% and 20.8%, respectively (both acids have the ability to chelate metal ions). The results of microbiological tests show that the material significantly inhibits the growth of *Aspergillus niger* and *Escherichia coli*. It has been observed that the modified material with silver ions causes complete inhibition of *A. niger* growth (after 48 h) and 98.6% for *E. coli* (after 24 h), while the use of peanut shells containing nanometric silver particles stops the growth of *Aspergillus niger* at level of ~ 88% after 48 h and *Escherichia coli* bacteria at level of ~ 89% after 24 h. This proves the very good antimicrobial properties of the material after the sorption process. The obtained results give the possibility of further research into possible use in practice.

## Electronic supplementary material

ESM 1 (DOCX 14 kb)
