# Supplementary material for: Sorption behavior of Arachis hypogaea shells against Ag+ ions and assessment of antimicrobial properties of the product
Source: Environ Sci Pollut Res Int. 2020 Mar 25;27(16):19530–42. doi: 10.1007/s11356-020-08464-2 (PMC7244469; doi:10.1007/s11356-020-08464-2)
Supplement: Supplementary file 1 — (DOCX 14 kb) [file 11356_2020_8464_MOESM1_ESM.docx]

Table 1S. Sorption of selected metal ions on peanut shells for variable pH (T = 20°C. C_0_ = 200 mg/dm^3^. w = 0.2 g)

| pH | Sorption capacity [mg/g] | | |
| --- | --- | --- | --- |
|  | Cu | Ag | Zn |
| No modification | 6.19 | 17.75 | 12.34 |
| 5 | 8.49 | 18.84 | 11.72 |
| 4 | 6.88 | 8.46 | 10.90 |
| 3 | 4.82 | 7.65 | 12.13 |
| 2 | 1.15 | 7.10 | 9.25 |
